# Mode of Rendering Sulphate of Quinine Tasteless

**Published:** 1849-02

**Authors:** 


### MODE OF RENDERING SULPHATE OF QUININE TASTELESS.

Dr. John Hardin, of Greensburg, Ky., informs us that he has been in the habit, for some time past, of administering sulphate of quinine in an infusion of slippery elm, and finds it the most eligible method he has ever tried. It is easy to invest the powder completely in the thick mucilage, and thus deprive it of taste, which is an important point where the patient is a child.

